# Spatiotemporal trends of pelvic organ prolapse incidence in North American swine breeding herds and association with climatic factors

**DOI:** 10.3389/fvets.2026.1779401

**Published:** 2026-03-05

**Authors:** Kimberly Aguirre Siliezar, José Pablo Gómez Vázquez, Rebecca Robbins, Maria Jose Clavijo, Beatriz Martínez-López

**Affiliations:** 1 Department of Medicine and Epidemiology, School of Veterinary Medicine, University of California, Davis, Davis, CA, United States; 2 Center for Animal Disease Modeling and Surveillance, Department of Medicine and Epidemiology, School of Veterinary Medicine, University of California, Davis, Davis, CA, United States; 3 Pig Improvement Company, Hendersonville, TN, United States; 4 Department of Veterinary Diagnostic and Production Animal Medicine, Iowa State University, Ames, IA, United States

**Keywords:** descriptive epidemiology, environmental risk factors, North America, POP, swine health

## Abstract

Sow mortality in the U. S. has nearly doubled, rising from 5.68% in 1997 to 14.5% in 2022. Recent studies cite sudden death, prolapses, lameness, PRRSV outbreaks, trauma, and infections as key contributors. Among these, pelvic organ prolapse (POP)—including uterine, vaginal, and rectal prolapses—has become a major global cause of sow mortality and morbidity. However, contemporary research on POP in North America is limited. This study addresses this gap by analyzing POP incidence in North American swine farms between 2019 and 2024 to (1) describe the spatiotemporal distribution of POP incidence, (2) identify significant clusters of increased POP incidence in space and time, and (3) evaluate potential associations between climatic factors (e.g., temperature, atmospheric moisture, wind speed, soil moisture) and POP incidence. Descriptive statistics were used to characterize trends, SaTScan was utilized for cluster analysis, and a zero-inflated beta regression model assessed associations between POP incidence and environmental factors. Results revealed a rising trend in POP across over 100 farms in the U. S. and Canada from 2019 to 2024, with consistently higher incidence occurring during colder months. We identified two significant clusters (*p* = 0.001) where POP incidence was higher within the cluster compared to outside. One cluster spanned five states over nearly 4 years (mean inside = 0.059 vs. mean outside = 0.029), suggesting widespread system-level risk, while the other was localized to a single farm (mean inside = 0.34 vs. mean outside = 0.032), reflecting potential farm-level drivers. Year and decreased soil moisture were significantly (*p* < 0.001) associated with increased POP incidence in the beta regression analysis. These findings highlight important patterns of POP in breeding herds, while offering valuable insights to help swine producers and veterinarians assess risk to sows and develop targeted mitigation strategies.

## Introduction

Studies over the past few decades have shown an increase in sow mortality, both globally and within North America (1, 2). Specifically looking at the U. S., a study published in 2000 found the mean annual mortality risk in 1997 to be 5.68% (3). Breeding herd data from PigCHAMP shows the yearly mean sow-mortality rate in the U. S. has steadily increased from 8.36% in 2013 to 14.73% in 2023 (4). We also see a similar increase in sow mortality in Canada, with rates increasing from 6.94 to 14.94% from 2013 to 2023 (4). For the year 2024, PigCHAMP combined data from the U. S. and Canada (labeled “North America”) and showed a mean sow-mortality rate of 12.51% (4). Sow mortality is caused by various factors, including heart issues, gastrointestinal problems, and reproductive disorders (5, 6). Recent studies highlight sudden death, prolapses, and lameness as significant contributors, followed by open pen gestations, trauma from infighting, and infections such as porcine reproductive and respiratory syndrome virus (PRRSV), Senecavirus A, and *Mycoplasma hyopneumoniae* (7–9).

One cause of morbidity and mortality that is becoming more concerning across swine farms globally is pelvic organ prolapse (POP), encompassing uterine, vaginal, and rectal prolapses. In 2019, a study looking at 155,238 sows from 144 swine herds in Spain determined that almost 1% of sows were removed due to prolapses, with an annual incidence rate of 3.8 prolapse cases per 1,000 sow-years (6). Another study published in Brazil identified different types of prolapses, specifically uterine, rectal, and bladder. The authors found that 5.7% of sows were diagnosed with uterine prolapse, 4% had rectal prolapse, and 1.6% had bladder prolapse (10). Other studies found higher rates of prolapse, with one 2019 study finding that uterine prolapses were diagnosed in 12.5% of sows and the most frequent condition diagnosed in their study sample (5).

The increasing prevalence of prolapses in sows seems to be the case in North America as well. As early as 1991, a Canadian study found that 6.6% of deaths in commercial sows were due to uterine prolapses (11). A 2007 case report from a North Carolina swine farm found that although the total number of sows with prolapses was low, prolapse was still one of the most prevalent conditions associated with non-sudden death during gestation (18.2%) and euthanasia during lactation (33.0%) stages (12). Another study looking at sow mortality records between 2009 and 2018 in four Midwestern U. S. farms noted that the percentage of deaths due to vaginal, uterine, or rectal prolapse ranged between 10 and 15% each year, but with no significant increase over time (1). More recently, a 2023 study looking at a swine production facility in the Midwest found that approximately 28% of sow mortality was attributed to prolapses (7).

Extensive research has been done on sow physiology and anatomy as it relates to POP. POP can be defined as the protrusion of normally internal pelvic structures, such as the vagina, uterus, and rectum, outside of the body (13). Sows have become increasingly susceptible to POP in recent years, which may be attributed to increased physiological pressure on sows. This pressure likely stems from genetic selection pressures, which select for things such as larger birth weights, leading to larger offspring and more potential complications during the birthing process (3). Some studies have found that physiological factors such as poor body condition, constipation, greater protrusion of the perineal area, having a long and flaccid uterus, and excessive relaxation of the pelvic and perineal region increase susceptibility of POP in sows (10, 14, 15). The vaginal microbiome and changes in its composition has also been identified as a potential indicator of risk for POP in sows (16, 17). Infectious agents [e.g., porcine reproductive and respiratory syndrome virus (PRRSV), porcine circovirus type 2 (PCV2), porcine parvovirus (PPV)] and environmental toxins (e.g., mycotoxins) have been associated with immunosuppression and reproductive issues (18–20), which may predispose sows to prolapse. However, the pathophysiology of POP is poorly understood in swine and livestock species in general, resulting in limited prevention options. Currently, culling or euthanasia of POP-affected sows is common practice in the swine industry and, as a result, incurs substantial economic losses for swine producers if enough of their herd is afflicted.

While the available literature has noted an increase in the prevalence of POP and its impact on sow health, we still know very little about the baseline prevalence of this condition in North America, the seasonal trends and spatiotemporal patterns of its occurrence, and its potential association with climatic, environmental, and management factors. To the best of our knowledge, there have been few to no studies looking at the spatio-temporal trends of POP in North America, or potential climatic predictors of POP. This study addresses this gap by analyzing POP incidence in over 100 North American swine breeding herds between 2019 and 2024. Specifically, we aim to: (1) describe the spatiotemporal distribution of POP incidence, (2) identify significant clusters of increased POP incidence in space and time, and (3) evaluate potential associations between climatic factors (e.g., temperature, atmospheric moisture, wind speed, soil moisture) and POP incidence.

## Materials and methods

### Data

The dataset utilized in this analysis was provided by industry collaborators, comprising >100 swine farms across the US and Canada that reported POP between 2019 and 2024. Farms recorded new POP cases, which were then used to calculate the monthly incidence for that farm (i.e., number of new POP cases/total number of at-risk sows on a specific farm for the corresponding month). Each observation in the dataset corresponded to a single farm’s incidence report for a given month-year. The POP report was submitted to the research team as an Excel file, and included information on POP incidence (pre-calculated as a proportion), site type, location (city, state, country), and time (month, year) of reporting. A categorical variable for season was generated according to the months in the dataset: Fall (September–November), Winter (December–February), Spring (March–May), Summer (June–August). Additionally, GPS coordinates for all farms were provided by industry collaborators and utilized for the analyses. Given that the dataset was derived from industry reports rather than random sampling, this represents a convenience sample of sow farms affiliated with a unique genetic company. While animals share a common genetic line, they originate from multiple suppliers rather than a single source farm. In addition, the sows were housed on different farms operated independently by various producers, with housing and management practices that may vary across locations. Importantly, the company provides the genetic material but does not oversee or participate in the care or management of the animals.

### Cluster analysis

A space–time analysis was conducted using SaTScan, allowing for the identification of clusters of high incidence of POP on swine farms (21). A normal probability model was ultimately chosen based on the outcome variable (i.e., POP incidence) being numerical and continuous. A Poisson probability model was considered; however, it was not chosen as the final model, given that the outcome variable in this case was not an integer.

Several spatial and temporal window combinations were assessed to evaluate the sensitivity of the results to changing assumptions. For the final analysis, a maximum spatial cluster size of 50% of the population at risk was selected, along with a maximum temporal window of 50% of the study period. No geographical overlap was selected as a criterion for reporting hierarchical clusters, to avoid redundancy. All other parameters were kept at default settings.

### Association with climatic factors

The climatic factors of interest in this study were: temperature, atmospheric moisture, soil moisture, and wind speed. These variables were chosen due to their potential to influence conditions associated with POP, including thermal stress, altered hydration status, constipation, dusty housing conditions leading to respiratory irritation and coughing, and mycotoxin growth leading to poor quality or moldy feed. These conditions may increase physiological strain and intra-abdominal pressure in sows, potentially increasing their risk of POP.

Beta regression models with a logit link were used to evaluate the association between POP incidence and certain environmental factors, specifically average temperature, atmospheric moisture (proxy for humidity), soil moisture (based on water volume in the upper soil layer), and horizontal wind speed calculated using eastward (u) and northward (v) wind speed components using the formula: $\sqrt{u^{2}+v^{2}}$ . Environmental data was downloaded from the ERA5-Land Monthly Aggregated—ECMWF Climate Reanalysis dataset from the Earth Engine Catalog (22).

Since the outcome variable (POP incidence) was bounded between 0 and 1 and included a high proportion of zero values, a zero-inflated beta regression model was deemed appropriate. The zero-inflated beta regression analysis produces two sets of results: a conditional model and a zero-inflated model. The conditional model estimates the association between the predictors and the incidence of POP given that the outcome is not a structural zero (i.e., actual cases of POP are occurring). In contrast, the zero-inflated model estimates associations between predictors and the likelihood that an observation is a structural zero, meaning that POP cases are truly absent rather than simply unobserved or unreported. Together, these two models can tell us which factors are associated with the presence/absence and magnitude of POP incidence.

Models were fitted using the glmmTMB package in RStudio (23). Univariate analysis was used to assess the association between each factor and POP incidence. Six different models, each varying in the number and type of predictors, were constructed and compared. Fixed effects included: season, year, temperature, atmospheric moisture, soil moisture, and wind speed. Location variables (e.g., city, state, country, longitude, latitude) were excluded from the model due to having too many levels that would overwhelm the model. A random intercept was created using the farm number to account for clustering of observations. Model performance and fitness were evaluated using Akaike Information Criterion (AIC), Bayesian Information Criterion (BIC), and log-likelihood values. Higher log-likelihood values and lower AIC and BIC values indicated better model fit.

## Results

### Spatiotemporal distribution

**Figure 1:**
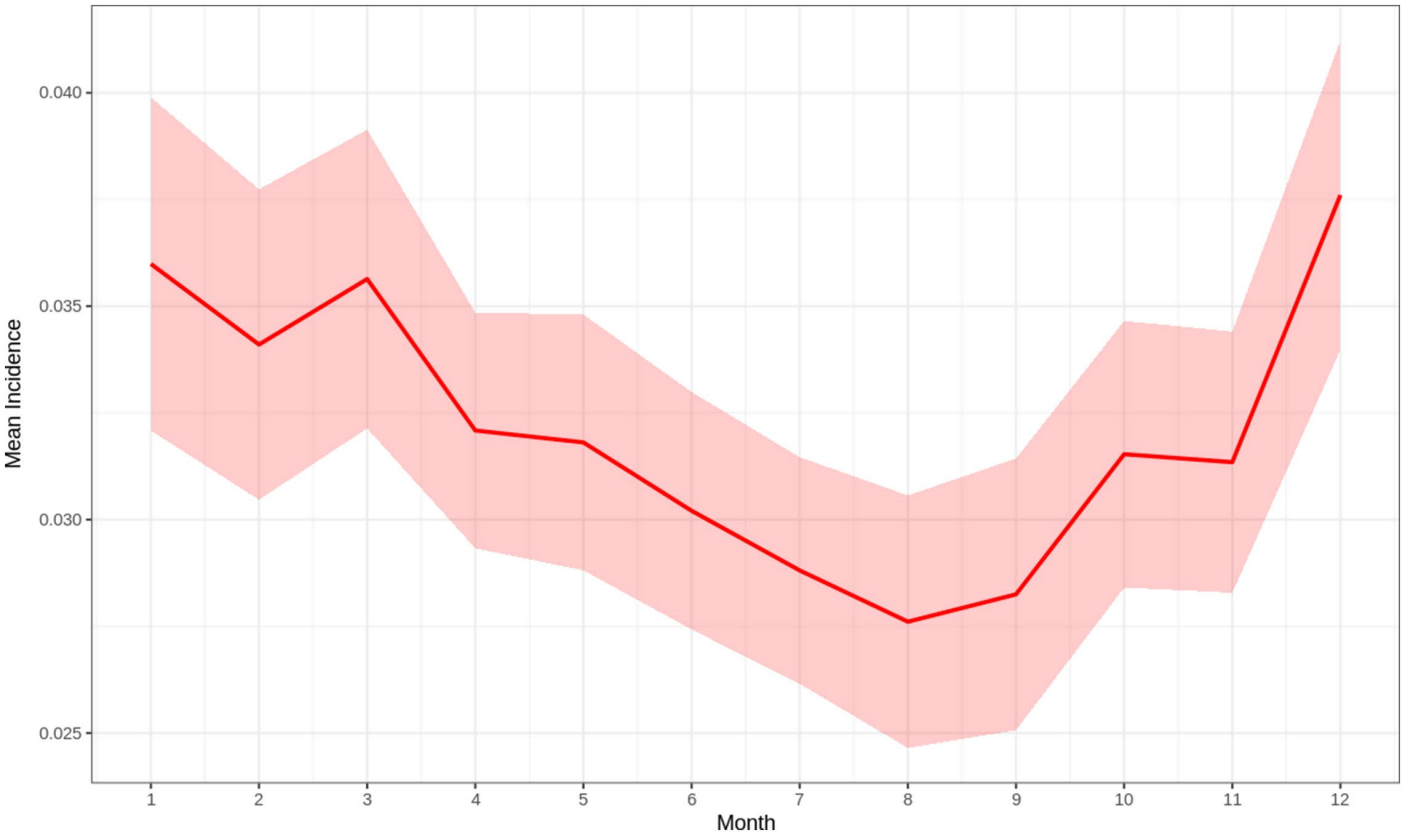
Aggregated mean incidence of pelvic organ prolapse in North American sows by month, 2019-2024. The red line represents the mean incidence per month, and the shaded ribbon denotes the 95% confidence interval.

**Figure 2:**
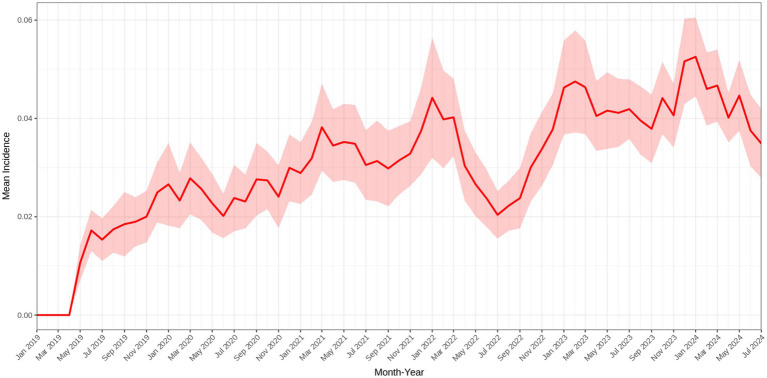
Time series for mean incidence of pelvic organ prolapse in sows in North America, 2019-2024. The red line represents the mean incidence per month-year, and the shaded ribbon denotes the 95% confidence interval.

**Figure 3:**
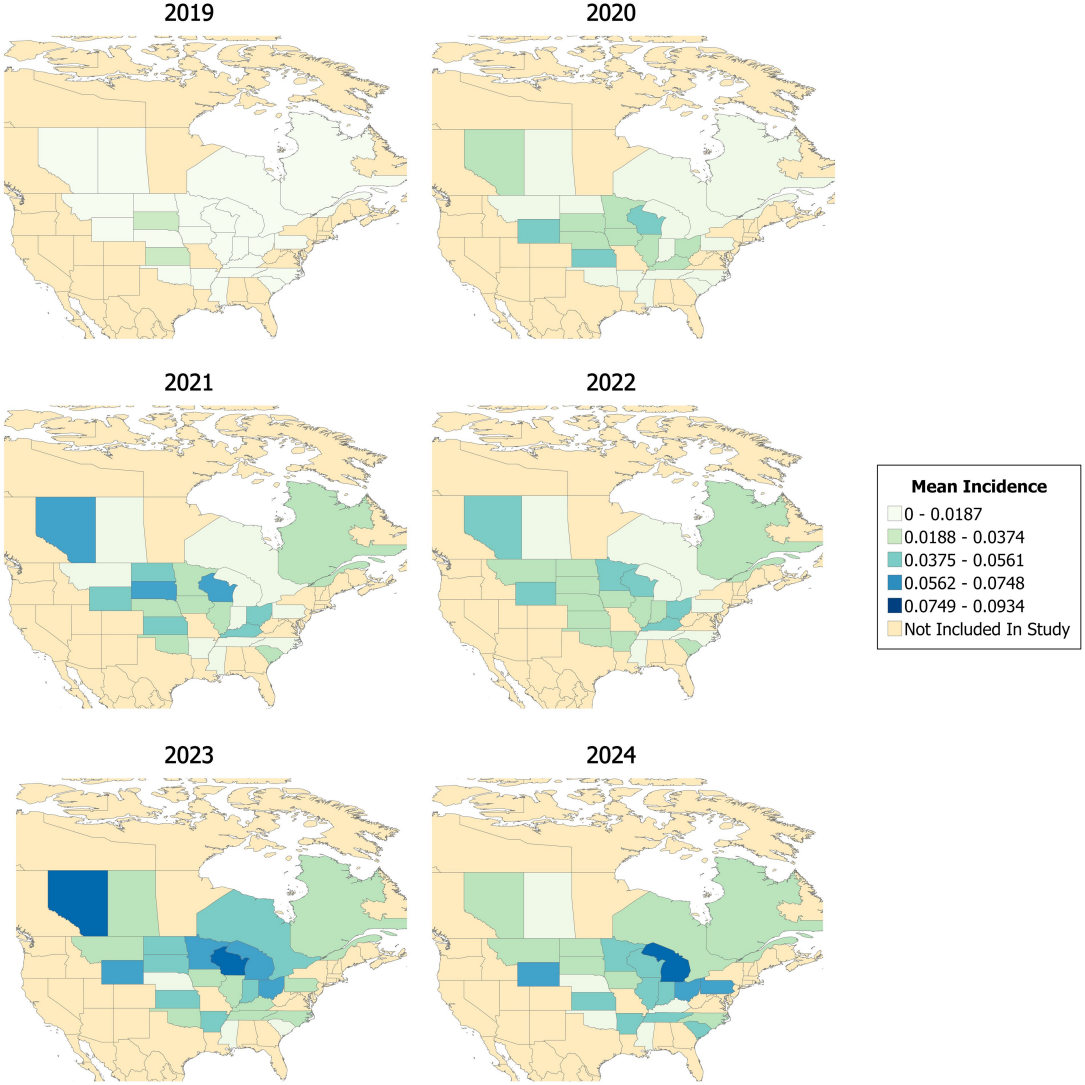
Mean annual incidence of pelvic organ prolapse in sows by state, 2019–2024. Color intensity reflects the average incidence per state per year, with darker shades indicating higher incidence rates. States not included in the study are shown in beige.

A total of 5,782 observations were analyzed across >100 different farms that reported POP between 2019 and 2024. Farms were located across 96 different cities and 25 states. Exploration of the data revealed a potential seasonal pattern, with POP incidence being highest in colder months (December through March) and lowest in warmer months (June through October) (Figure 1). POP incidence overall appeared to increase over time (Figures 2, 3). Two major peaks occurred in late 2021 and early 2024, as well as a notable drop in POP incidence in mid-2022 (Figure 2). Increased incidence of POP appears to expand geographically over time across both Canadian and U. S. states. Between 2021 and 2024, the central Midwest, along with Montana, Iowa, Wisconsin, and Alberta, appeared to be consistent areas for high POP incidence (Figure 3). Some states maintained consistently low POP incidence (Mississippi) or appeared to decrease (Nebraska) over time (Figure 3).

### Cluster analysis

Only two clusters of increased POP incidence were identified as being statistically significant (*p* = 0.001). One cluster occurred between October 2022 to July 2024, and involved 673 reports of POP across 31 unique farms from 7 different states (Iowa, Illinois, Indiana, Michigan, Ohio, Wisconsin, US, and Ontario, Canada). The mean POP incidence inside this cluster was found to be twice as high as the mean POP incidence outside (inside: 0.059, outside: 0.029). The second cluster consisted of only one farm in Quebec, Canada, between November 2021 and February 2022. The mean POP incidence was approximately 10 times greater inside the cluster compared to outside (inside: 0.34, outside: 0.032). Six other clusters were identified in the analysis, but none were statistically significant.

### Association with environmental factors

The final model chosen based on best fit contained season, year, temperature, atmospheric moisture, soil moisture, and wind speed as fixed effects. The zero-inflated beta regression analysis produced two sets of results, one for the conditional model and the other for the zero-inflated model.

In the conditional model, year and soil moisture were significant predictors (Table 1). All years from 2020 to 2024 showed a higher likelihood of POP incidence being non-zero compared to 2019. Estimates for each year increased over time, with the largest increase being in 2024 (estimate: 0.607, 95% CI: 0.524–0.691, *p* < 0.001). Soil moisture also had a higher probability of POP incidence being non-zero [estimate: −0.701, 95% CI: −1.232(−0.170), *p* = 0.01], suggesting that as soil moisture levels decreased, POP cases were more likely to occur. No significant association was found between season or other environmental variables and POP incidence after excluding structural zeros.

**Table 1:** Conditional model results from the zero-inflated beta regression analysis.

Conditional model				
Predictor	Estimate	Std. error	*p*-value	95% CI
Intercept	−1.755	0.593	**0.003**	(−2.916, −0.591)
Year: 2020	0.188	0.043	**<0.001**	(0.104, 0.272)
Year: 2021	0.453	0.041	**<0.001**	(0.373, 0.534)
Year: 2022	0.451	0.042	**<0.001**	(0.369, 0.534)
Year: 2023	0.592	0.040	**< 0.001**	(0.513, 0.671)
Year: 2024	0.607	0.043	**< 0.001**	(0.524, 0.691)
Season: spring	0.029	0.029	0.318	(−0.028, 0.086)
Season: summer	−0.026	0.033	0.441	(−0.091, 0.039)
Season: winter	0.030	0.036	0.400	(−0.040, 0.101)
Temperature	−0.010	0.007	0.149	(−0.023, 0.004)
Atmospheric moisture	0.004	0.007	0.535	(−0.009, 0.018)
Soil moisture	−0.701	0.271	**0.010**	(−1.232, −0.170)
Wind speed	0.015	0.018	0.394	(−0.020, 0.050)

Bolded values denote statistical significance (*p*-value < 0.05).

In the zero-inflated model results (Table 2), year and soil moisture were again found to be significant, along with season and other environmental factors. All years after 2019 were associated with significantly lower odds of POP incidence truly being zero. Estimates for each year decreased over time, with the largest decrease in 2024 [estimate: −1.732, 95% CI: −1.968(−1.496), *p* < 0.001]. Increases in soil moisture (estimate: 2.817, 95% CI: 1.837–3.797, *p* < 0.001) and summer (estimate: 0.400, 95% CI: 0.189–0.612, *p* < 0.001) were associated with higher odds of structural zeros (i.e., POP cases less likely to be observed under these conditions). Winter [estimate: −0.392, 95% CI: −0.622(−0.162), *p* < 0.001], increased wind speed [estimate: −0.256, 95% CI: −0.372(−0.139), *p* < 0.001] and increased temperature [estimate: −0.059, 95% CI: −0.095(−0.022), *p* 0.002] were associated with lower odds of structural zeros (i.e., POP cases are less likely to be truly absent under these conditions).

**Table 2:** Zero-inflated model results from the zero-inflated beta regression analysis.

Zero-inflated model				
Predictor	Estimate	Std. error	*p*-value	95% CI
Intercept	7.007	1.582	**<0.001**	(3.907, 10.107)
Year: 2020	−1.051	0.106	**<0.001**	(−1.258, −0.843)
Year: 2021	−1.167	0.104	**<0.001**	(−1.371, −0.963)
Year: 2022	−0.848	0.099	**<0.001**	(−1.042, −0.654)
Year: 2023	−1.612	0.105	**<0.001**	(−1.819, −1.406)
Year: 2024	−1.732	0.120	**<0.001**	(−1.968, −1.496)
Season: spring	0.023	0.093	0.806	(−0.160, 0.206)
Season: summer	0.400	0.108	**<0.001**	(0.189, 0.612)
Season: winter	−0.392	0.117	**<0.001**	(−0.622, −0.162)
Temperature	−0.059	0.019	**0.002**	(−0.095, −0.022)
Atmospheric moisture	0.033	0.020	0.101	(−0.006, 0.072)
Soil moisture	2.817	0.500	**<0.001**	(1.837, 3.797)
Wind speed	−0.256	0.060	**<0.001**	(−0.372, −0.139)

Bolded values denote statistical significance (*p*-value < 0.05).

## Discussion

This study revealed important temporal and geographic patterns in POP incidence among sows in North American commercial swine systems. The observed seasonal trend—with incidence peaking in winter months and dropping in summer—suggests a possible environmental or management-related influence on POP risk. Extreme cold-weather stress, changes in feed composition/consumption, co-infections (e.g., PRRS), seasonal management changes (e.g., ventilation adjustments), or physiologic demands during colder months could contribute to this pattern, although further investigation is needed to support this hypothesis.

The overall upward trend in POP incidence over the six-year study period is concerning and supports the current body of literature on POP in sows. This increase may reflect either a true rise in incidence or changes in reporting practices over time (e.g., increased surveillance and reporting). Two clear spikes in incidence—late 2021 and early 2024—were speculated to reflect outbreak-like events, broader systemic changes (e.g., nutrition, genetics, or disease pressure), changes in market standards, or increased reporting. The transient dip in mid-2022 was unexpected and puzzling. Further data exploration found that the number of farms contributing data in 2022 was among the highest recorded across the study period, and that monthly reporting frequency remained consistent with other years. This suggests that the decline in 2022 is less likely due to under-reporting and more likely reflects short-term epidemiologic or management-related factors (e.g., herd demographics, disease pressures, short-term interventions). Without detailed information at the sow and farm-level during this year, we cannot determine the precise cause of the decline.

The identification of only two statistically significant spatiotemporal clusters reinforces the hypothesis that POP is not evenly distributed across time and space. Both clusters varied greatly in timespan, with one occurring over almost 4 years and the other over 4 months. These clusters also varied spatially, with one spanning a wide geographic area, indicating that POP may affect diverse production systems in different regions. Notably, the small but intense cluster affecting a single farm—with a mean POP incidence over 10 times the background incidence—warrants further investigation into farm-level risk factors. Ultimately, these findings suggest that geographic and temporal clustering may be less informative than other factors.

The zero-inflated beta regression results supported the observed upward trend in POP incidence over time, but the association with environmental factors was not as significant as expected. Year and soil moisture were found to be significant predictors in both the conditional and zero-inflated models (*p* < 0.001), indicating that POP incidence steadily increased from 2020 to 2024 and with decreased levels of soil moisture. While we did not directly measure soil conditions, farm-level sanitation, or feed contamination, soil moisture may plausibly influence POP risk indirectly. For example, soil moisture may promote fungal growth on crops and lead to mycotoxin contamination in feed, a process that can have a delayed effect on overall sow health (24, 25). Additionally, even indoor-housed sows may be exposed to dust via foot traffic, equipment, or ventilation, which can contribute to respiratory stress or coughing. Although these mechanisms remain speculative, they provide a biologically plausible explanation for the observed associations and highlight the need for further research on direct effects of feed quality and housing sanitation practices on POP incidence. In contrast, seasonal and climatic factors were only significant in the zero-inflated model. Specifically, summer months were associated with higher odds of POP incidence being a true zero (POP genuinely absent), whereas winter, increased temperatures, and increased wind speed were linked with reduced odds of true zeros (POP less likely to be truly absent). This pattern indicates that season, temperature, wind speed were associated with the probability of POP occurring, rather than the magnitude of incidence when cases are present. These findings suggest a weaker or less consistent relationship between these variables and POP incidence compared to year and soil moisture.

This study builds on a 2018 study from the Iowa Pork Industry Center in collaboration with Iowa State University, which surveyed 104 swine breeding herds across multiple states in the U. S. to identify potential risk factors for POP (15). They reported significant associations between POP risk and perineal scoring, bump feeding, body condition scores during late gestation, and water quality. While this survey did not assess climatic or seasonal factors, our results suggest that environmental conditions (soil moisture) may contribute to POP risk. These findings highlight POP as a multifactorial disease process that is influenced by both individual-level management changes and broader environmental conditions, resulting in increased physiologic strain in sows. Further studies combining management and environmental data could clarify how these factors interact with POP and one another to drive POP risk.

There were several limitations in this study. Reporting bias may have been present, considering that some farms may have been more likely than others to report POP, leading to possible underestimation or overestimation of the true POP incidence. There was also inconsistency in the data quality across participating farms and over time, which could be attributed to reporting and recording practices, variability in how POP cases were defined, and completeness of the data. Another major limitation was that the data were collected at the farm level rather than at the sow level, which restricted our ability to assess individual-level risk factors such as body condition, reproductive stage, parity, litter size, or genetic susceptibility. These factors have been well-established in the literature as determinants of POP, and may also be correlated with environmental or farm-level management variables. However, the absence of this individual-level data limits causal inference since we cannot discern whether observed associations are a reflection of direct, farm-level factors or unmeasured individual-level characteristics. Differences in environmental and management factors across the farms also present a limitation in this study. Factors such as nutrition, biosecurity protocols, welfare, and handling practices may influence POP risk but were not captured in this dataset, which may confound the results presented here. Given that all sow farms have a common genetic supplier, this genetic relatedness may limit the generalizability of our findings to farms using other genetic suppliers. In regard to the spatial analysis of this dataset, this may have been subject to bias since farms were not randomly distributed, and differences in participation may have caused overrepresentation of some regions and underrepresentation of others. Lastly, the dataset did not provide information on the different types (e.g., vaginal, uterine, rectal) or severity of POP. This makes it challenging to identify patterns in POP that could help inform more clinically specific interventions.

Overall, these results suggest that temporal trends and production system characteristics may be more influential than geographic clustering and environmental factors in predicting POP risk. While soil moisture was found to be significant, this may act as a proxy for other environmental or management-related risk factors, such as mycotoxins. Our findings highlight the need for seasonal surveillance and staff training to aid in early recognition of POP, which will help enhance welfare and reduce sow losses. Future research efforts should focus on integrating sow and farm-level data to identify biological, management-related, and other potential risk factors.

## Data Availability

The datasets presented in this article are not readily available because of the sensitive nature of the data. Access to the data is restricted to authorized individuals and institutions in compliance with the agreed-upon terms. De-identified datasets may be shared upon request. Requests to access the datasets should be directed to Beatriz Martínez-López, beamartinezlopez@ucdavis.edu.
